# Plasma Extracellular Vesicle-Derived TIMP-1 mRNA as a Prognostic Biomarker in Clear Cell Renal Cell Carcinoma: A Pilot Study

**DOI:** 10.3390/ijms21134624

**Published:** 2020-06-29

**Authors:** Francisca Dias, Ana Luísa Teixeira, Inês Nogueira, Mariana Morais, Joana Maia, Cristian Bodo, Marta Ferreira, Isabel Vieira, José Silva, João Lobo, José Pedro Sequeira, Joaquina Maurício, Jorge Oliveira, Carlos Palmeira, Gabriela Martins, Klaas Kok, Bruno Costa-Silva, Rui Medeiros

**Affiliations:** 1 Molecular Oncology and Viral Pathology Group, IPO-Porto Research Center (CI-IPOP), Portuguese Oncology Institute of Porto (IPO-Porto), Research Center- LAB2, E Bdg 1st floor, Rua Dr António Bernardino de Almeida, 4200-072 Porto, Portugal; Francisca.Carvalho.Dias@ipoporto.min-saude.pt (F.D.); inescmnogueira@gmail.com (I.N.); mariana.gomes.morais@ipoporto.min-saude.pt (M.M.); ruimedei@ipoporto.min-saude.pt (R.M.); 2 Institute of Biomedical Sciences Abel Salazar, University of Porto (ICBAS-UP), Rua Jorge Viterbo Ferreira 228, 4050-513 Porto, Portugal; 3 Research Department of the Portuguese League Against Cancer Regional Nucleus of the North (LPCC-NRN), Estrada da Circunvalação 6657, 4200-177 Porto, Portugal; 4 Systems Oncology Group, Champalimaud Research, Champalimaud Centre for the Unknown, Av. Brasília, 1400-038 Lisbon, Portugal; joana.maia@research.fchampalimaud.org (J.M.); cristian.bodo@research.fchampalimaud.org (C.B.); bruno.costadasilva@research.fchampalimaud.org (B.C.-S.); 5 Graduate Program in Areas of Basic and Applied Biology (GABBA), University of Porto, 4200-135 Porto, Portugal; 6 Department of Medical Oncology, Portuguese Oncology Institute of Porto (IPO-Porto), Rua Dr António Bernardino de Almeida, 4200-072 Porto, Portugal; marta.ribeiro.ferreira@ipoporto.min-saude.pt (M.F.); jmauricio@ipoporto.min-saude.pt (J.M.); 7 Department of Urology, Portuguese Oncology Institute of Porto (IPO-Porto), Rua Dr António Bernardino de Almeida, 4200-072 Porto, Portugal; isabel.vieira@ipoporto.min-saude.pt (I.V.); silvajfg@gmail.com (J.S.); jorge.oliveira@ipoporto.min-saude.pt (J.O.); 8 Department of Pathology, Portuguese Oncology Institute of Porto (IPO-Porto), Rua Dr António Bernardino de Almeida, 4200-072 Porto, Portugal; jpedro.lobo@ipoporto.min-saude.pt; 9 Cancer Biology and Epigenetics Group, IPO-Porto Research Center (CI-IPOP), Portuguese Oncology Institute of Porto (IPO-Porto), Research Center- LAB3, F Bdg 1st floor, Rua Dr António Bernardino de Almeida, 4200-072 Porto, Portugal; jose.leite.sequeira@ipoporto.min-saude.pt; 10 Department of Pathology and Molecular Immunology, Institute of Biomedical Sciences Abel Salazar, University of Porto (ICBAS-UP), Rua Jorge Viterbo Ferreira 228, 4050-513 Porto, Portugal; 11 Department of Immunology, Portuguese Oncology Institute of Porto (IPO-Porto), Rua Dr António Bernardino de Almeida, 4200-072 Porto, Portugal; carlospalmeira@ipoporto.min-saude.pt (C.P.); gmartins@ipoporto.min-saude.pt (G.M.); 12 Experimental Pathology and Therapeutics Group, IPO-Porto Research Center (CI-IPOP), Portuguese Oncology Institute of Porto (IPO-Porto), Research Center- LAB2, E Bdg 1st floor, Rua Dr António Bernardino de Almeida, 4200-072 Porto, Portugal; 13 Faculty of Health Sciences, Fernando Pessoa University (UFP), Praça 9 de Abril 349, 4249-004 Porto, Portugal; 14 Department of Genetics, University Medical Center Groningen (UMCG), University of Groningen, Hanzeplein 1, 9713 GZ Groningen, P.O. Box 30.001, 9700 RB Groningen, The Netherlands; k.kok@umcg.nl; 15 Faculty of Medicine, University of Porto (FMUP), Alameda Prof. Hernâni Monteiro, 4200-319 Porto, Portugal

**Keywords:** clear cell Renal Cell Carcinoma, Extracellular Vesicles, TIMP-1, mRNA

## Abstract

The tumor microenvironment has gained a lot of attention from the scientific community since it has a proven impact in the development of tumor progression and metastasis. Extracellular vesicles (EVs) are now considered one of the key players of tumor microenvironment modulation. Clear cell renal cell carcinoma (ccRCC) is the most lethal urological neoplasia and presents a high metastatic potential, which reinforces the need for the development of more effective predictive biomarkers. Our goal was to evaluate the applicability of EV-derived matrix metalloproteinases (MMPs) and tissue inhibitors of metalloproteinases (TIMPs) as prognostic biomarkers for ccRCC. To do so, we studied the plasma EV content of 32 patients with localized ccRCC and 29 patients with metastatic ccRCC. We observed that patients with localized disease and tumors larger than 7 cm presented higher levels of plasma EV-derived TIMP-1 mRNA when compared with patients presenting smaller tumors (*p* = 0.020). Moreover, patients with metastatic disease presented higher levels of EV-derived TIMP-1 mRNA when compared with patients with localized disease (*p* = 0.002) and when we stratified those patients in high and low levels of TIMP-1 EV-derived mRNA, the ones presenting higher levels had a lower overall survival (*p* = 0.030). EV-derived TIMP-1 mRNA may be a good prognostic biomarker candidate for ccRCC.

## 1. Introduction

Cancer is a heterogeneous disease, with different etiology and natural history, that develops through interactions between environmental and genetic factors, involving the deregulation of multiple pathways responsible for the fundamental cell processes, such as proliferation, differentiation, migration and cell death [1]. However, despite the existing knowledge of several genetic factors on tumor pathophysiology, understanding the complex molecular mechanisms underlying its development remains unclear, implying that, besides the intrinsic malignant properties of tumor epithelial cells, other factors such as microenvironmental changes may modulate tumor progression, invasion and metastasis [2]. One way of microenvironment shaping is through paracrine and/or systemic signaling among cells that can be made through the release of soluble factors or through the shedding of extracellular vesicles (EVs). There are several classes of EVs, with microvesicles and exosomes considered the most important in terms of biomolecules transport between cells. Microvesicles are formed by the outward budding of the plasma membrane of the cell while exosomes originate from multivesicular bodies and are released from cells when the multivesicular bodies fuse with the cell surface [3]. In the vast repertoire of bioactive molecules that EVs carry, we can find proteins, DNA and several classes of RNAs including non-coding RNAs, such as microRNAs, mRNAs, rRNAs and tRNAs [4]. EVs are able to shuttle bioactive molecules between cells and their cargo can induce pro-tumorigenic tumor microenvironments, locally or at distant sites, since EVs can travel around the body through the bloodstream [5]. Thus, phenotypic alterations induced by incorporation of EVs by recipient cells may precede metastatic dissemination, since EVs can have an impact on extracellular matrix (ECM) remodeling through the delivery of several molecules, including functional matrix metalloproteinases (MMPs) and tissue inhibitors of metalloproteinases (TIMPs), or their mRNAs, into target cells [6,7,8,9,10].

MMPs are a family of 23 zinc-dependent endopeptidases that are crucial for ECM degradation and the processing of cell surface molecules [11]. On the other hand, the TIMP family, which is composed of four elements, is responsible for the regulation of the pericellular proteolysis of ECM and cell surface proteins through inhibition of MMPs [11]. Both MMPs and TIMPs are often deregulated in cancer with an impact on patients’ prognosis [12,13]. Regarding their incorporation into EVs, to date 11 MMPs and three TIMPs have been identified in EVs derived from various cell types, including cancer cells, but the available information is still very limited [14]. Since the presence of MMPs and TIMPs in EVs can have implications in ECM remodeling and, consequently, in the modulation of the structural architecture and dynamics of the tumor microenvironment, both locally and at distant sites, it is of paramount importance to increase the current knowledge on their impact on cancer progression.

Renal cell carcinoma (RCC) is the most common solid cancer of the adult kidney and also the most lethal urological cancer [15]. The most common and aggressive subtype is the clear cell RCC (ccRCC), which accounts for approximately 80% of all RCCs [16]. The increased use of routine imaging techniques during the past decades led to the incidental detection of early stage localized RCCs, while the patients were doing exams for other medical reasons. However, due to the kidneys anatomical location, many tumors remain asymptomatic until the late stages of disease [16]. In fact, one-third of RCC patients present metastatic disease at diagnosis and 20–40% of patients submitted to nephrectomy will present local recurrence or distant metastasis [17]. CcRCC is chemo- and radio-resistant and although systemic treatment with targeted therapies may improve patients’ survival, the development of resistance is very common [18]. Thus, there is an urgent need for the establishment of accurate prognostic and predictive biomarkers in order to improve the follow-up of these patients. The aim of this study was the establishment of a plasma EV-derived MMP/TIMP mRNA profile with potential to be used as a prognosis biomarker for ccRCC.

## 2. Results

### 2.1. EVs Characterization

**Figure 1:**
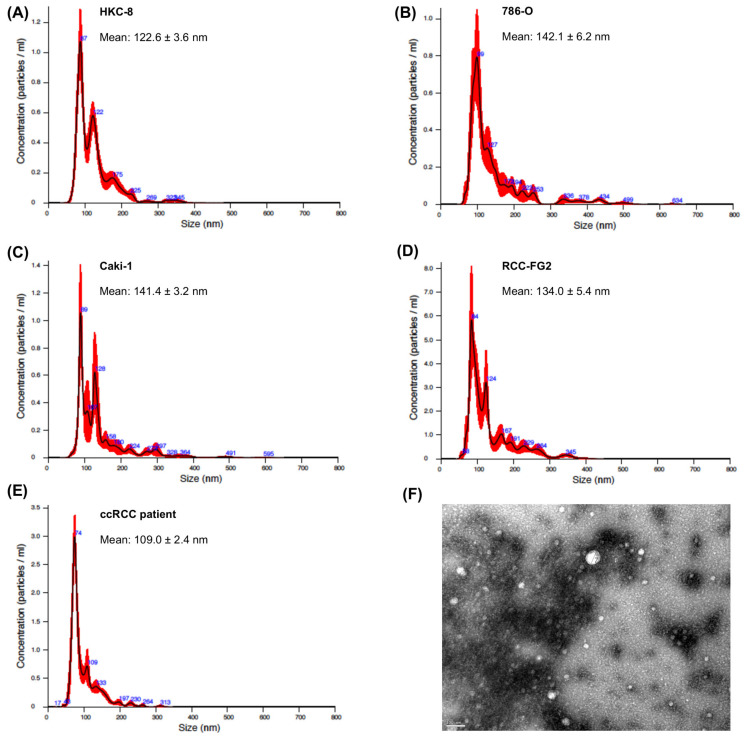
Nanoparticle tracking analysis (NTA) of extracellular vesicles (EVs) derived from (**A**) HKC-8, (**B**) 786-O, (**C**) Caki1 and (**D**) RCC-FG2 cells and (**E**) clear cell renal cell carcinoma (ccRCC) patients. The red error bars indicate ± 1 standard error of the mean. (**F**) Transmission electron microscopy (TEM) of EVs from purified platelet-free plasma (PFP). The TEM image was acquired in the Histology and Electron Microscopy platform from I3S Porto using a Transmission Electron Microscope Jeol JEM 1400.

EVs were isolated from HKC-8, 786-O, Caki-1 and RCC-FG2 cell lines and from the ccRCC patients included in the study. The Nanoparticle Tracking Analysis (NTA) indicated that the vast majority of isolated EVs presented a size range between 50 and 200 nm, which is consistent with the size of exosomes and small microvesicles (Figure 1A–E). The Transmission Electron Microscopy (TEM) image (Figure 1F) shows the variability of sizes and morphology present in EVs from purified platelet-free plasma (PFP). We also utilized EVs Flow Cytometry to confirm the purity of our EVs isolates. This method uses Carboxyfluorescein Diacetate Succinimidyl Ester (CFSE) staining to differentiate vesicular from non-vesicular particles, as this dye only becomes fluorescent when incorporated and processed within vesicles, and has been described by some authors as a pan-EV label [19,20,21]. In all cases, approximately 80% of particles present in our isolates corresponded to CFSE^+^ vesicular structures (Figure S1).

### 2.2. MMP-1, TIMP-1 and TIMP-2 Present Different Protein Levels among Renal Cell-Derived EVs

**Figure 2:**
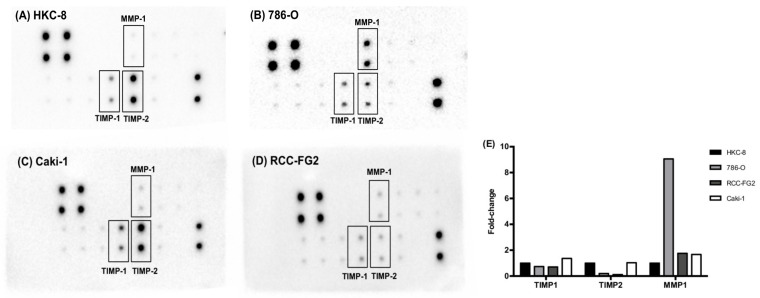
Human MMP Antibody Array analysis in cell-derived EVs. Membranes were probed with EV lysate from (**A**) HKC-8, (**B**) 786-O, (**C**) Caki-1 and (**D**) RCC-FG2 cells. MMP, matrix metalloproteinases; TIMP, tissue inhibitor of metalloproteinase; Pos, positive control; Neg, negative control. (**E**) Fold-change of the pixels volume adjusted intensity.

In order to evaluate the MMPs and TIMPs protein content in EVs derived from HKC-8, 786-O, Caki-1 and RCC-FG2 cell lines we did a multiplexed MMP/TIMP array. The MMP/TIMP array map is displayed in Table 1. Compared to the normal kidney cell line HKC-8 (Figure 2A), all the three ccRCC-derived cell lines diverged in terms of EV protein expression: the 786-O cell line (B) presented a higher expression of MMP-1 and a lower expression of TIMP-2, the Caki-1 cell line (C) presented similar expression in terms of TIMP-2 and a slightly higher of TIMP-1, and the RCC-FG2 cell line (D) presented lower expression of TIMP-2 and similar expression of TIMP-1. Interestingly, the expression of MMP-1 was higher in the 786-O cell line (B), which is derived from a primary ccRCC tumor, when compared to both metastatic ccRCC cell lines (C and D). In addition, TIMP-1 and TIMP-2 expression were also different in both ccRCC metastatic cell lines (C and D), with the Caki-1 cell line presenting higher levels of TIMP-1 and TIMP-2. Taken together, these data suggest that different ccRCC clones may present different MMP/TIMP profiles.

### 2.3. MMP-1, TIMP-1 and TIMP-2 mRNA Levels in Renal Cell Lines and Cell Line-Derived EVs

**Figure 3:**
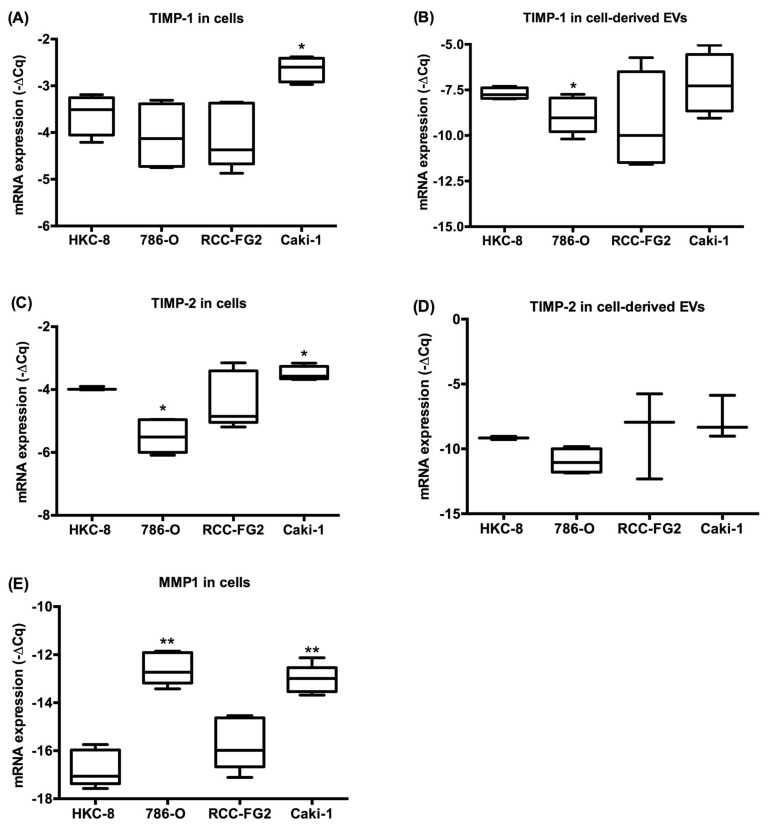
TIMP-1, TIMP-2 and MMP-1 mRNA expression intracellularly (**A**, **C** and **E**) and in EVs (**B** and **D**) derived from HKC8, 786-O, RCC-FG2 and Caki-1 cell lines. (Mean ± Std. Error; * *p* < 0.05, ** *p* < 0.001). Five biological replicates of each cell line were used for this experiment.

After the characterization of the EV-derived MMP/TIMP protein content of HKC-8, 786-O, RCC-FG2 and Caki-1 cell lines, we quantified the mRNA levels of MMP-1, TIMP-1 and TIMP-2 inside the cells and in the cell-derived EVs. The Caki-1 cell line presented the highest intracellular TIMP-1 mRNA expression compared to the other three cell lines (Figure 3A). Regarding mRNA expression in the EVs, TIMP-1 was detected in all EV fractions. The 786-O cell line presented lower TIMP-1 expression when compared to the HKC-8, and the Caki-1 cell line presented a trend for higher TIMP-1 mRNA than the 786-O cell line (*p* = 0.089) (Figure 3B). As for TIMP-2, HKC8 and Caki-1 had the highest intracellular mRNA levels and 786-O presented the lowest, which is in agreement with the MMP/TIMP array results (Figure 3C). TIMP-2 mRNA was detected in all EV fractions without any statistical differences in the expression level between them (Figure 3D).

The 786-O cell line presented the highest MMP-1 intracellular mRNA levels when compared to the other cell lines, which is in agreement with the results of the MMP/TIMP array (Figure 3E) However, the Caki-1 cell line also showed elevated MMP-1 mRNA expression which was not reflected in protein expression in the MMP/TIMP array. The mRNA levels of MMP-1 in cell-derived EV’s are not presented, since they were only detected in the EV’s derived from the 786-O cell line, and absent in the EVs from the other cell lines.

### 2.4. TIMP-1, TIMP-2 and MMP-1 mRNA Expression in ccRCC Patients’ EVs

**Figure 4:**
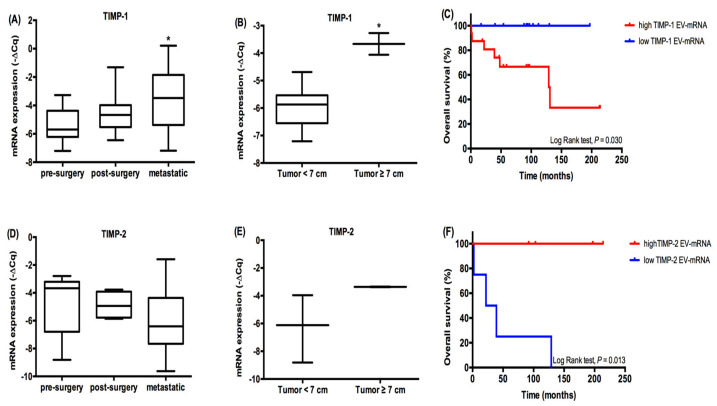
EV-derived TIMP-1 and TIMP-2 mRNA expression in ccRCC plasma samples. EV-derived TIMP-1 (**A**) and TIMP-2 (**D**) mRNA expression in patients with localized disease (pre- and post-surgery) and metastatic disease. EV-derived TIMP-1 (**B**) and TIMP-2 (**E**) mRNA expression in patients with localized with tumor < 7 cm and ≥ cm. Overall survival of metastatic ccRCC patients according to EV-derived TIMP-1 (high expression levels *n* = 16, low expression levels *n* = 11) (**C**) and *TIMP-2* (high expression levels *n* = 4, low expression levels *n* = 4) (**F**); mRNA expression (Mean ± Std. Error; * *p* < 0.05, ** *p* < 0.001).

After the detection and quantification of the mRNA levels of TIMP-1, TIMP-2 and *MMP-1* in the cell lines and cell-derived EVs, we proceeded to the quantification of the same mRNAs in the EVs derived from plasma samples of ccRCC patients. We observed that patients with metastatic disease presented higher *TIMP-1* EV mRNA levels compared to patients with localized disease (*p* = 0.002) (Figure 4A). When we focused on the patients with localized disease, we evaluated the EV-derived mRNA levels in patients with tumors smaller than 7 cm versus patients with tumors larger than 7 cm due to the fact that, according to the AJCC TNM, 7 cm is the limit size between stage I and stage II patients. Therefore, by grouping the patients in tumors smaller than 7 cm versus patients with tumors larger than 7 cm, we will be evaluating the EV-derived mRNA levels in initial stage patients (stage I) versus higher stage patients (stage II, III and IV). We observed that those with tumors larger than 7 cm also presented higher levels of EV-derived TIMP-1 mRNA (*p* = 0.020) (Figure 4B). Next, we divided the metastatic patients into high and low levels of TIMP-1 EV-derived mRNA using the −ΔCq mean as a cutoff value. We observed that patients with metastatic disease and higher levels of TIMP-1 EV-derived mRNA presented a lower overall survival compared to patients presenting with lower levels (Log Rank test, *p* = 0.030) (Figure 4C).

There were no statistically significant differences for TIMP-2 EV-mRNA levels between patients with localized versus metastatic disease (*p* = 0.338) nor in terms of tumor size in the patients presenting localized disease (*p* = 0.477) (Figure 4D,E). When we focused on the survival of metastatic patients, those presenting lower EV-derived TIMP-2 mRNA levels had a lower overall survival (Log Rank test, *p* = 0.013) (Figure 4F). However, it is important to note that TIMP-2 EV-derived mRNA was only detected in eight samples, and these results must be interpreted with caution.

Regarding the other clinical pathological characteristics, we observed that patients who smoke had higher levels of EV-derived TIMP-1 mRNA when compared to non-smokers (*p* = 0.028) and ex-smokers (*p* = 0.053). We did not observe any statistical differences between EV-derived TIMP-1 mRNA and hypertension (*p* = 0.957) or diabetes mellitus (*p* = 0.461). The low number of samples where EV-derived TIMP-2 mRNA was detected was not sufficient to allow any statistical analysis using these clinical pathological characteristics (data not shown). The same applies to MMP-1 EV-derived mRNA expression that was only detected in four samples in total (1 from Group A + 3 from Group B) and did not allow any statistical analysis (data not shown). The percentages of TIMP-1, TIMP-2 and MMP-1 mRNA detection in patients’ EVs are presented in Figure S2.

### 2.5. EV-Derived TIMP1 mRNA as a Prognostic Marker in ccRCC Patients: The Example of Two Patients

As stated previously, two groups of patients were analyzed in the current study: one group with localized disease whose blood samples were collected before and after surgery and another group already with metastatic disease. From the 32 patients included in the localized disease group (Group A), two of them developed metastasis during the follow-up period. Interestingly, both of them presented high levels of EV-derived TIMP-1 mRNA both in the pre- and post-surgery samples (when compared to the other patients with localized disease), and both of them underwent radical nephrectomy. The patients’ clinical data are presented in Table 2. Given the fact that our survival analysis indicated that metastatic patients with higher EV-derived TIMP-1 mRNA present a lower overall survival and that these two patients with elevated EV-derived TIMP-1 mRNA developed metastatic disease within a short period of time, we can hypothesize that the levels of EV-derived TIMP-1 mRNA could be a prognosis biomarker, namely a metastatic disease predictor.

Taking this into account, we further investigated the clinical data of the ccRCC metastatic patients that presented high levels of TIMP-1 mRNA and are currently deceased from the disease. From the seven patients that were deceased, three of them presented metastasis at the moment of diagnosis and the remaining four presented localized disease when they were diagnosed but passed away during a five-year period. These four patients presented at least two recurrences each during this period, which is consistent with a more aggressive disease.

## 3. Discussion

MMPs are one of the major classes of proteolytic enzymes involved in tumor invasion and metastasis establishment and their action can be modulated by TIMPs. Despite earlier studies having demonstrated that TIMPs had antimetastatic effects, during the last few years, reports started to indicate a dual function of these proteins, with a positive correlation between their expression and a poorer outcome in several human cancers [22,23,24,25,26]. Recent studies of protein profiles have revealed the presence of TIMPs in EVs from various cell types, but their biological impact remains to be elucidated [14]. TIMP-1 and TIMP-2 proteins have been found in EVs derived from cancer cells, bone marrow mesenchymal stem cells and also in pregnant women [27,28,29]. In terms of their potential impact in cancer, it is known that EV-derived TIMP-1 binds to CD63 and β1 integrin, which induces survival signals and promotes metastatic niche formation [11]. EV-derived TIMP-2, on the other hand, plays a crucial role in the formation of the MT1-MMP-TIMP-2-pro-MMP-2 ternary complex for *MMP-2* activation, which is well known for its impact on cancer progression [30,31].

In order to get more insight into the EV-derived MMP/TIMP profile in ccRCC, we started with the study of EVs derived from four different renal cell lines. All of the cell lines exhibited different EV-derived MMP/TIMPs protein profiles, with the most prominent differences in the expression of MMP-1, TIMP-1 and TIMP-2. These results highlighted the importance of the intratumor heterogeneity in ccRCC, since different ccRCC clones presented different EV-derived MMP/TIMP profiles and may have different impact on the disease evolution. An example of that was what we observed for the two metastatic ccRCC cell lines, Caki-1 and RCC-FG2, were the Caki-1 cells presented higher protein expression of TIMP-1 and TIMP-2 than RCC-FG2.

Next, we focused on the mRNA levels of MMP-1, TIMP-1 and TIMP-2 intracellularly and in the shedded EVs from all the cell lines studied, in order to see if there were also differences in the expression patterns. We observed that TIMP-1 and TIMP-2 mRNAs were present in all the cell lines EVs whereas *MMP-1* mRNA was detectable in 786-O EVs. It is important to note that mRNA molecules are known to be unstable and prone to degradation and their incorporation into EVs is described as the only mechanism that allows their stable circulation in body fluids without the risk of degradation by RNase activity, making them very attractive molecules to study in circulating EVs [32]. We further studied the EV-derived mRNA expression of TIMP-1, TIMP-2 and MMP-1 mRNAs in plasma EVs from two groups of ccRCC patients: patients with localized disease before and after tumor removal surgery (Group A) and patients with metastatic disease (Group B). In patients with localized disease, those with larger tumors had higher expression of EV-derived TIMP-1 mRNA. When comparing both groups of patients (A versus B), we observed that patients with metastatic disease had higher expression of EV-derived TIMP-1 mRNA than patients with localized disease. In fact, metastatic patients presenting a higher expression of EV-derived TIMP-1 mRNA presented a lower overall survival. In addition, we observed that patients who smoke presented higher EV-derived TIMP-1 mRNA levels compared to non-smokers. The opposite effect was observed for EV-derived TIMP-2 mRNA, with lower expression levels being associated with a lower overall survival. However, TIMP-2 EV-derived mRNA was quite difficult to detect in ccRCC patients’ EVs (being detected only in a few patients), which suggests that it may not be a good biomarker candidate for ccRCC and the same applies to MMP-1 EV-derived mRNA.

During the course of follow-up, two of the patients with localized disease (group A) developed metastasis several months after surgery. When we looked up to the levels of EV-derived *TIMP-1* mRNA, we observed that both patients presented high levels before surgery and those levels remained high one month after surgery. Although they are speculative and require further validation, these results suggest that EV-derived TIMP-1 mRNA may play a role in disease progression, even when the primary tumor is removed. It is important to note that tumor growth and invasion are also supported by non-cancerous stromal cells, which include endothelial cells, fibroblasts, pericytes and immune cells, all of which are also able to secrete EVs [33,34,35]. These cell types are an active part of the tumor microenvironment, and several studies have already demonstrated that tumor-derived EVs are able to exert various effects on neighboring stromal cells, including the stimulation of EVs release [5,14,36,37]. One additional factor that may contribute to the increase of EVs enriched in TIMP-1 mRNA is cigarette smoking. Cigarette smoking is known to have a destructive effect on the ECM, as it leads to the recruitment of activated macrophages and neutrophils leading to an imbalance between MMPs and TIMPs [38]. It has already been reported that smoking is associated with an increase of TIMP-1 mRNA, and we observed that patients who smoke present higher levels of EV-derived TIMP-1 mRNA [39].

Furthermore, a study performed by Cui et al. demonstrated that an increase of both endogenous and exogenous levels of TIMP-1 led to upregulation of miR-210 in a CD63-PI3K-AKT-HIF1-dependent pathway in lung adenocarcinoma cells. Moreover, upon the overexpression of TIMP-1, miR-210 was accumulated in EVs that were able to promote angiogenesis, both in vitro and in xenograft models, once in contact with recipient cells, thereby evidencing the pro-tumorigenic function of TIMP-1 [40]. Our group has previously associated the high plasma levels of miR-210 with a worse prognosis in ccRCC patients, and this evidence could be linked to the higher circulating levels of *TIMP-1* EV-derived mRNA, but further studies are needed in order to validate this hypothesis [41]. In addition, in a recent study by our group regarding the potential use of plasma EV-derived miRNAs as prognostic biomarkers for ccRCC using the same groups of patients, we observed that EV-derived miR-1293 was downregulated in metastatic patients [42]. Since *TIMP-1* is one validated target of miR-1293, the decrease of miRNA levels observed in the EVs from the metastatic patients may be related to the upregulation of EV-derived *TIMP-1* mRNA observed in the present study; however, further studies are needed in order to validate this hypothesis.

Despite being a pilot study involving a small group of patients, this study was able to acknowledge the potential of using EV-derived mRNAs as potential circulating biomarkers in cancer. In addition, systemic mRNA delivery, although still in its infancy, holds a huge potential for application in cancer vaccination and immunotherapy since its advantages over DNA transfection make it attractive in applications where transient expression is desired [43]. The mRNA stability conferred by its incorporation in EVs may help in the development of future nano-based therapeutics with applications in several types of disease [43,44,45].

To the best of our knowledge, this is the first study to address the potential of *TIMP-1* EV-derived mRNA as a prognosis biomarker for ccRCC patients with metastatic disease. Future studies involving larger cohorts of patients, with an increased follow-up period, are needed in order to further validate our hypothesis.

## 4. Materials and Methods

### 4.1. Study Design

**Figure 5:**
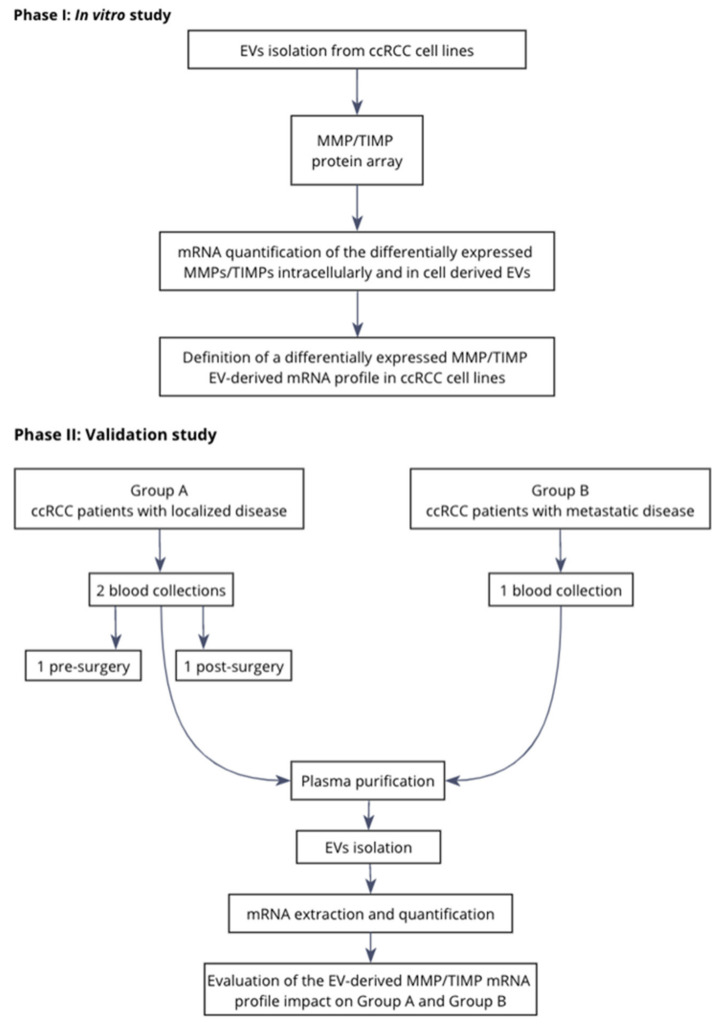
Study design flow chart.

The present study was divided in two phases, one in vitro study and one validation study, and was conducted according to the flow chart represented in Figure 5.

### 4.2. Ethics Statement

This study was conducted according to the principles of the Helsinki declaration, having been approved by the ethics committee of the Portuguese Oncology Institute of Porto (project reference: CES 251/015; approval date: 12 November 2015). All individuals have signed written informed consent in order to participate in the study.

### 4.3. Study Population

The analysis of the EV-derived TIMP-1, TIMP-2 and MMP-1 mRNA expression was made through a hospital-based study involving a total of 61 ccRCC patients. All individuals were Caucasian from the north of Portugal, with histopathological diagnosis of ccRCC, admitted and treated at the Portuguese Oncology Institute of Porto (IPO-Porto) between November 2015 and June 2019. The patients were divided into two groups: Group A consists of 32 patients diagnosed with localized disease that were going to undergo surgical intervention; and Group B consists of patients already presenting metastatic disease. The clinical data described in Table 2 are the data collected at the moment of diagnosis. Thus, it is important to note that, from 29 patients of group B, 20 developed metastatic disease during the follow-up period after surgery and seven were already diagnosed with metastatic disease. Clinical characteristics of patients were obtained from their medical records (Table 3). Patients from Group A collected blood twice, before undergoing surgery and approximately 1–3 months after surgery, and patients from Group B collected blood once. All blood collections were performed during the morning period.

Tumor classification and staging were established according to the tumor-node-metastasis (TNM) classification system of the American Joint Committee on Cancer (AJCC) 8th edition (2018) and the International Society of Urological Pathology (ISUP) Classification of Renal Neoplasia [46].

### 4.4. EVs Isolation

EVs were isolated from the cell-culture medium using the Total Exosome Isolation Reagent (from cell culture media) (Invitrogen^TM^, Waltham, MA, USA) with additional protocol optimizations. Briefly, cell medium was collected and centrifuged 30 min at 2000 g to remove cells and debris. After centrifugation, the supernatant was recovered and filtered through a 0.22 μm filter (GE Healthcare Whatman ^TM^, Chicago, IL, USA). Then the clarified medium and the exosome isolation reagent were mixed, in a proportion of 2:1, and incubated overnight at 4 °C. Afterwards, the mix was centrifuged 1 h at 10,000 g and the pellet containing the pre-enriched EVs was resuspended in filtered PBS and stored at −80 °C until further analysis.

Regarding the peripheral blood samples, EVs were isolated from the plasma fraction using the Total Exosome Isolation Kit from Plasma (Invitrogen^TM^, Waltham, MA, USA) with additional protocol optimizations. Firstly, 8 mL of peripheral blood was collected from the patients in EDTA tubes and centrifuged 5 min at 1800 g to obtain the plasma fraction. The plasma fraction was centrifuged three additional times at increasing speeds (300 *g*, 2100 *g* and 10,000 *g*) for a period of 15 min each in order to obtain platelet-free plasma (PFP). After centrifugation, the supernatant was recovered and filtered through a 0.22 μm filter (GE Healthcare Whatman ^TM^, Chicago, IL, USA). A 10-min treatment with proteinase K, the Total Exosome Isolation (TEI) reagent was added to 200 μL of PFP and the solution was incubated for 30 min at 4 °C. The precipitated EVs were recovered by a 5 min centrifugation at 10,000 *g* at room temperature. The pellet containing the pre-enriched EVs was resuspended in filtered PBS (0.22 µm membrane filters) and stored at −80 °C until further analysis.

### 4.5. EVs NTA Analysis

All samples were analyzed for particle concentration and size distribution by the NS300 Nanoparticle Tracking Analysis (NTA) system (NanoSight—Malvern Panalytical, Malvern, UK). Samples were pre-diluted in filtered PBS to achieve a concentration within the range for optimal NTA analysis. Video acquisitions were performed using a camera level of 16 and a threshold between 5 and 7. Five to nine videos of 30 s were captured per sample. Analysis of particle concentration per mL and size distribution was performed with NTA software v3.4 (Malvern Panalytical, Malvern, UK).

### 4.6. Quantification of Vesicular Structures by EVs Flow Cytometry

We employed EVs flow cytometry for quantification of vesicular structures in our EV isolates, as recently described by Maia et al. [47]. In total, 2 × 10^9^ particles of purified EVs were mixed with 40 µL of PBS containing Carboxyfluorescein Diacetate Succinimidyl Ester (CFSE—Thermo Fisher Scientific—LTI C34554, Waltham, MA, USA) in a final concentration of 40 µM and incubated for 90 min at 37 °C. For removal of unbound CFSE, Size Exclusion Chromatography (SEC) columns (iZON—qEV original columns SP1, Oxford, UK) were used. Samples containing unstained or stained EVs, and appropriate controls, were diluted up to 500 µL of PBS and processed by qEV following manufacturer’s instructions. EVs-enriched fractions #7, #8 and #9 were then compiled and retrieved for analysis with the Flow Cytometer Apogee A60-Micro-Plus (Apogee Flow Systems, London, UK). The A60-Micro-Plus machine is equipped with three spatially separated lasers (488 nm—Position C, 405 nm—Position A and 638 nm—Position B), seven fluorescence color detectors (525/50, LWP590, 530/30, 574/26, 590/40, 695/40, 676/36) and three light scatter detectors (SALS, MALS and LALS). For internal control across assays, before each FC experiment, we used two mixes of beads (Apogee—1493 and Apogee—1517, Apogee Flow Systems, London, UK). Before being loaded, samples were diluted in filtered PBS (0.22 µm membrane filters) to bring their concentration within the operational range of the equipment (maximum of 3000 events/s). All samples were run at a flow rate of 1.5 µL/min using a 405 nm—LALS threshold of 70. The 405 nm—LALS PMT noise level was monitored and always maintained below 0.35. For the experiments depicted, the stopping criteria utilized was the number of events acquired, so samples were run until a minimum of 250,000 events was reached. The acquired data was exported and analyzed with FlowJo software v10.4.2 (FlowJo LLC, Ashland, OR, USA).

### 4.7. Protein Extraction and Multiplexed MMP Array

The pre-enriched EVs and cell pellets were diluted in PBS, 1X RIPA lysis buffer. Halt^TM^ Protease & Phosphatase Inhibitor cocktail (Thermo Scientific^®^, Waltham, MA, USA) were added, and the samples were incubated at 4 °C for 15 min. Protein was quantified using the BCA protein assay (BioRad^®^, Hercules, CA, USA). Human MMP Antibody Array (ab134004, Abcam^®^, Cambridge, UK) was used to measure MMPs and TIMPs. This ELISA-like multiplex approach offers substantial benefits for examining a defined set of proteins in parallel and detects seven human MMPs (MMP-1, MMP-2, MMP-3, MMP-8, MMP-9, MMP-10 and MMP-13) and three TIMPs (MMP-13, TIMP-1, TIMP-2 and TIMP-4). Activity was determined in EVs lysates using 100 ng of protein input. Quantification was performed by ChemiDoc^TM^ XRS+ System and data analysis was performed using Quantity One^®^ Analysis Software (BioRad^®^, Hercules, CA, USA).

### 4.8. RNA Extraction and cDNA Synthesis

RNA isolation and purification of the cells and cell-derived EVs was done using the RNeasy Plus Mini Kit (Qiagen^®^, Hilden, Germany) according to the manufacturer procedure. RNA concentration and purity were measured using the NanoDrop Lite spectrophotometer (Thermo Scientific^®^, Waltham, MA, USA) and served as a template for cDNA synthesis using a High Capacity cDNA Reverse Transcription Kit (Applied Biosystems^®^, Foster City, CA, USA)). The thermal conditions for the cDNA synthesis were as follows: 25 °C for 10 min followed by 37 °C for 120 min and 85 °C for 5 min.

### 4.9. Quantitative Real Time PCR

mRNA expression levels were analyzed by quantitative real-time PCR. The reactions were carried out in a StepOnePlus^TM^qPCR Real-Time PCR machine, in a volume of 10 ul containing 1X TaqMan^TM^ Fast Advanced Master mix (Applied Biosystems, Foster City, CA, USA), with 1X TaqMan^TM^ mRNA Expression Assays probes (TIMP-1: Hs99999139_m1; TIMP-2: Hs00234278_m1 and MMP-1: Hs00899658_m1—Applied Biosystems, Foster City, CA, USA), and cDNA. For mRNA expression normalization two housekeeping controls were used: GAPDH (Hs02758991_g1—Applied Biosystems, Foster City, CA, USA) and ACTB (Hs01060665_g1—Applied Biosystems, Foster City, CA, USA). These housekeeping genes were chosen based on the fact that they are reported as typical EVs cargo [48]. The amplification conditions were as follows: holding stage 95 °C for 20 s, followed by 45 cycles of 95 °C for 1 s and 60 °C for 20 s. Three technical replicates were made for each sample. Data analysis was done using StepOne^TM^ Sofware v2.2 (Applied Biosystems, Foster City, CA, USA) with the same baseline and threshold set for each plate, in order to generate quantification cycle (Cq) values for all the mRNAs in each sample.

### 4.10. Statistical Analysis

Statistical analyses were done using IBM SPSS Statistics software for Windows Version 22.0 (IBM^®^, Armonk, NY, USA). According the mRNAs levels distribution, the Student t-test or Mann Whitney U test were used in order to evaluate any statistical differences in the normalized expression of the mRNAs. The quality of the housekeeping genes was tested using the BestKeeper software [49]. Both endogenous controls presented a stable behavior, so both were used to normalize the mRNAs levels. The Kaplen Meier method and Log Rank test were used to establish the association of the *TIMP-1* derived EV mRNA levels (low versus high) to the overall survival. The *TIMP-1* derived EV mRNA levels low versus high levels were defined using the mean value of the –ΔCq.

## Figures and Tables

**Table 1:** MMP/TIMP Antibody Array map.

	A	B	C	D	E	F	G	H
1	Pos	Pos	Neg	Neg	MMP-1	MMP-2	MMP-3	MMP-8
2	Pos	Pos	Neg	Neg	MMP-1	MMP-2	MMP-3	MMP-8
3	MMP-9	MMP-10	MMP-13	TIMP-1	TIMP-2	TIMP-4	Neg	Pos
4	MMP-9	MMP-10	MMP-13	TIMP-1	TIMP-2	TIMP-4	Neg	Pos

**Table 2:** Clinical characteristics of the two patients that present high levels of EV-derived *TIMP-1* mRNA in the pre- and post-surgery samples.

	Gender	Age	Tumor Size	TNM Stage	ISUP	Time to Recurrence	Metastatic Site
*Patient A*	male	83	6 cm	pT3aNxM0R0	4	9 months	lung
*Patient B*	male	74	8 cm	pT3cN0M0R0	3	21 months	bones

**Table 3:** Clinical-pathological characteristics of the study population. Group A consists of patients with localized disease and Group B consists of patients with metastatic disease.

		Group A (*n* = 32)		Group B (*n* = 29)	
		*n*	%	*n*	%
*Gender*					
	Male	24	75.0	21	72.4
	Female	8	25.0	8	27.6
*Age*	Average ± SD	61.9 ± 12.4		62.4 ± 9.9	

*Type of surgery*					
	Partial nephrectomy	18	56.3	0	0
	Radical nephrectomy	14	43.8	27	93.1
	No surgery	0	0	2	6.9
*Tumor size*					
	<7 cm	24	75.0	7	24.1
	≥7 cm	8	25.0	18	62.1
	Undetermined	0	0	4	13.8
*T*					
	T1	17	53.1	8	27.6
	T2	1	3.1	7	24.1
	T3	13	40.6	10	34.5
	T4	0	0	1	3.4
	Tx	1	3.1	3	10.6
*n*					
	N0	0	0	15	51.7
	N1-N2	0	0	3	10.3
	Nx	0	0	9	31.0
*M*					
	M0	32	100	20	68.9
	M1	0	0	7	24.1
	Mx	0	0	2	7.0
*Clinical stage*					
	I	17	53.1	7	24.1
	II	1	3.1	6	20.7
	III	13	40.6	8	27.6
	IV	0	0	5	17.2
	Not available	1	3.1	3	10.4
*ISUP classification*					
	1	2	6.3	1	3.45
	2	18	56.3	12	41.4
	3	11	34.3	7	24,1
	4	1	3.1	5	17.2
	Not available	0	0	4	13.8
*Smoking status*					
	Smoker	6	18.8	2	6.9
	Ex-smoker	11	34.4	4	13.8
	Non-smoker	14	43.8	23	79.3
	Not available	1	3.1	0	0
*Hypertension*					
	Yes	20	62.5	14	48.3
	No	11	34.4	15	51.7
	Not available	1	3.1	0	0
*Diabetes mellitus*					
	DM I	0	0	0	0
	DM II	9	28.1	12	41.4
	No	22	68.8	17	58.6
	Not available	1	3.1	0	0

## Supplementary Materials

Supplementary Materials can be found at https://www.mdpi.com/1422-0067/21/13/4624/s1.

## Abbreviations

ACTB	Actin Beta
AJCC	American Joint Committee on Cancer
AKT	AKT Serine/Threonine Kinase
ccRCC	Clear cell Renal Cell Carcinoma
CFSE	Carboxyfluorescein Diacetate Succinimidyl Ester
Cq	Quantification cycle
EMT	Epithelial Mesenchymal Transition
EVs	Extracellular Vesicles
GAPDH	Glyceraldehyde 3-phosphate dehydrogenase
HIF1	Hypoxia Inducible Factor 1
ISUP	International Society of Urological Pathology
MMP-1	Matrix metalloproteinase 1
NTA	Nanoparticle Tracking Analysis
PFP	Platelet-free-plasma
PI3K	Phosphatidylinositol 3-kinase
TIMP-1	Tissue inhibitor of metalloproteinase 1
TIMP-2	Tissue inhibitor of metalloproteinase 1
TNM	Tumor-node-metastasis
