# Supplementary material for: Plasma Extracellular Vesicle-Derived TIMP-1 mRNA as a Prognostic Biomarker in Clear Cell Renal Cell Carcinoma: A Pilot Study
Source: Int J Mol Sci. 2020 Jun 29;21(13):4624. doi: 10.3390/ijms21134624 (PMC7370073; doi:10.3390/ijms21134624)
Supplement: Supplementary file 1 [file ijms-21-04624-s001.pdf]

Supplementary Material

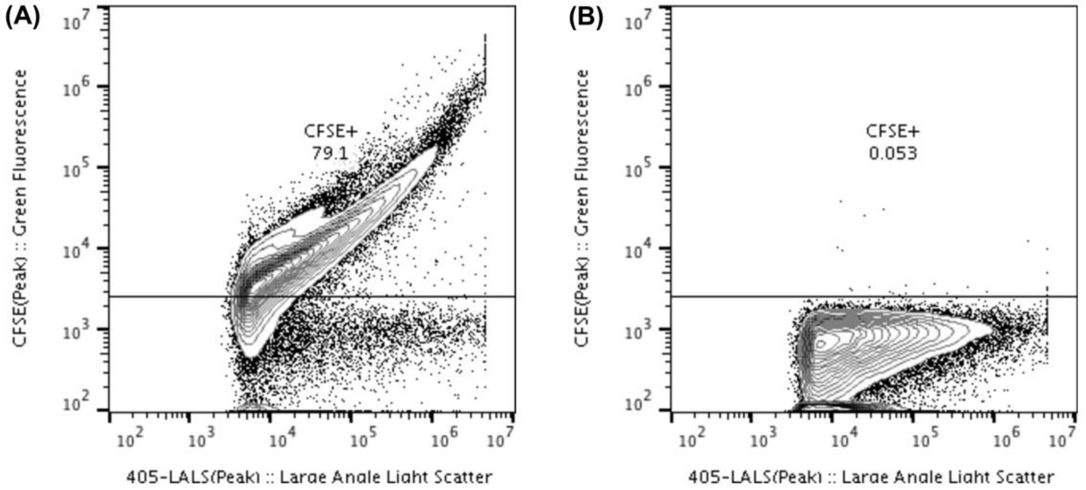


**Figure S1** - Proportion of fluorescente EVs as observed by flow cytometry from a sample of EV isolate derived from a patient, either previously stained with CFSE (A) or without previous staining (B).

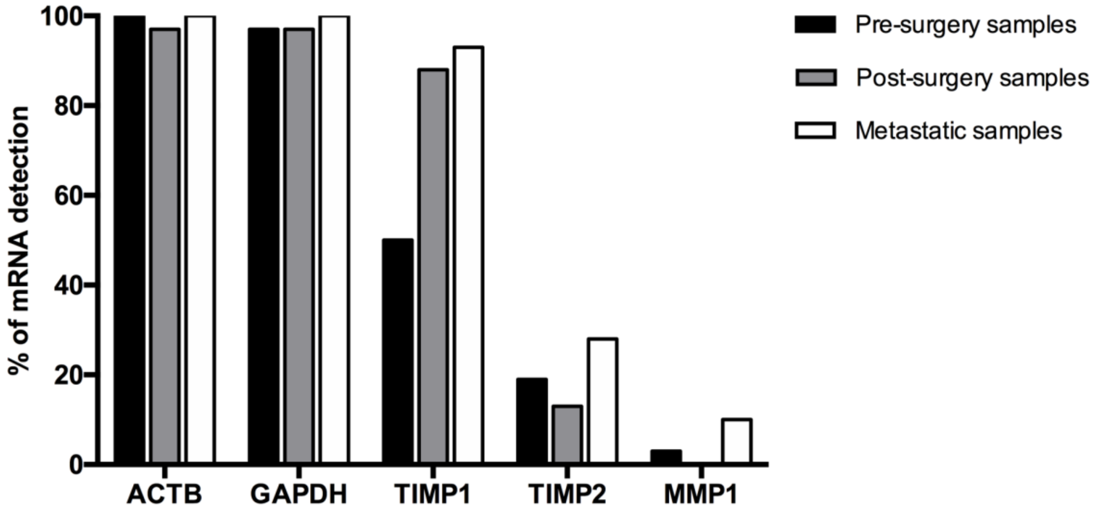


**Figure S2** - Percentages of *ACTB*, *GAPDH*, *TIMP-1*, *TIMP-2* and *MMP1* mRNA detection in EVs derived from ccRCC patients.
